# Short-Term Adverse Pregnancy Outcomes in Women with Subclinical Hypothyroidism: A Comparative Approach of Iranian and American Guidelines

**DOI:** 10.1155/2022/9315250

**Published:** 2022-03-05

**Authors:** Farahnaz Mir, Hossein Chiti, Saeideh Mazloomzadeh

**Affiliations:** ^1^Zanjan Metabolic Diseases Research Center, Zanjan University of Medical Sciences, Zanjan, Iran; ^2^Social Determinants of Health Research Center, Zanjan University of Medical Sciences, Zanjan, Iran

## Abstract

**Introduction:**

Subclinical hypothyroidism during pregnancy can be associated with numerous adverse outcomes. The purpose of this study was to compare short-term adverse pregnancy outcomes in treated versus nontreated patients who fall within the numerical range of thyroid-stimulating hormone (TSH) between the Iranian and American reference ranges.

**Materials and Methods:**

Eighty pregnant women with a known level of antithyroid peroxidase (anti-TPO) and TSH levels of 2.5–3.9 mIu/L in the first trimester and 3–4.1 mIu/L in the second and third trimesters were enrolled in the study and randomly assigned into two groups including 41 patients in the intervention group and 39 in the control group. The intervention group was treated with levothyroxine at least 50 *μ*g/day and the control group received no treatment. The data were analyzed by SPSS software version 23.

**Results:**

The only significant findings were a correlation between pregnancy loss frequency (*p* − 0.011) and/or increased TSH level in the follow-up period (*p* = 0.008) with anti-TPO antibody positivity. Forty-four percent of mothers with positive anti-TPO Ab needed treatment initiation with levothyroxine, based on Iranian guidelines, due to increased TSH level during the follow-up period.

**Conclusion:**

Untreated pregnant women with subclinical hypothyroidism, who were placed in the intermediate range of TSH, recommended by Iranian and American guidelines, did not show any significant difference in short-term adverse pregnancy outcomes compared to the treated patients. Positive anti-TPO Ab may play a role in the development of short-term complications in mothers with subclinical hypothyroidism or it may increase the likelihood of an increase in TSH level during pregnancy.

## 1. Introduction

Numerous comorbidities may threaten pregnancy, one of which is subclinical hypothyroidism (SCH) [1]. Iranian and American reference ranges have introduced different levels of TSH as a criterion for initiating treatment in SCH during pregnancy. According to Iranian guidelines, when the TSH level is higher than 3.9 mIu/ml in the first trimester or higher than 4.1 mIu/ml in the second and third trimesters of pregnancy, the risk of adverse pregnancy outcomes may increase [2]. American guidelines put these numbers above 2.5 mIu/ml in the first trimester and above 3 mIu/ml in the second and third trimesters [3]. The most important adverse outcomes of pregnancy resulting from untreated SCH are pregnancy loss [4], miscarriage [5,6], intrauterine fetal death [5], placental abruption [7], and an increased risk of neonatal cognitive-neurological disorders [8,9]. The information obtained from systematic reviews and meta-analysis is so limited which necessitates further studies in this field. An important meta-analysis in Iranian pregnant women showed a high prevalence of thyroid disorders especially subclinical and clinical hypothyroidism [1]. Another meta-analysis evaluated the complications of pregnancy in mothers with subclinical hypothyroidism and showed that these women were at a higher risk for pregnancy loss, neonatal death, placental abruption, and premature rupture of membranes compared to euthyroid women [10]. Several meta-analyses, albeit with a small number of studies, have reported the preventive effects of levothyroxine therapy on maternal and fetal complications in pregnant women with subclinical hypothyroidism. The most important maternal and fetal complications that have decreased following levothyroxine treatment in these women are pregnancy loss and neonatal death [11,12]. Most meta-analyses have not been able to show a beneficial effect of levothyroxine treatment in pregnant women with subclinical hypothyroidism in reducing other maternal and fetal complications or improving the IQ level of children resulting from these pregnancies [11–13]. In one of these meta-analyses, levothyroxine treatment even led to preterm labor [11]. Most studies also have revealed an association between thyroid autoimmunity and recurrent pregnancy loss [14]. Due to the possibility of these complications and lack of enough data, treatment of SCH in pregnancy actually seems a necessity. The Iranian guideline is based on a study conducted in Tehran, and given the ethnic differences in Iran, the need to launch other studies in other provinces seemed reasonable. On the other hand, the decision to treat mothers who had a TSH number between the numbers recommended by the Iranian guideline and the numbers recommended by the American guideline was not specified. In this study, we investigated the occurrence of adverse short-term outcomes of pregnancy, especially pregnancy loss, preterm delivery, and premature rupture of the membrane in untreated pregnant mothers who had a TSH number between the two recommended numbers proposed by the Iranian and American guidelines. The proposal of this article was approved by the Ethics Committee of Zanjan University of Medical Sciences and was registered on the IRCT site with the code: IRCT20180314039091N1.

## 2. Materials and Methods

In a randomized clinical trial, 106 pregnant women who primarily had a TSH level of ≥3.9 mIu/ml and ≤2.5 mIu/ml in the first trimester of pregnancy or ≥4.1 mIu/ml and ≤3 mIu/ml in the second and third trimesters of pregnancy, reevaluated with thyroid function tests (TSH, total T4, and free T4) and anti-TPO Ab in a predetermined laboratory and who again fulfilled the abovementioned TSH range, included in the study. The required sample size was calculated based on the available data about adverse pregnancy outcomes attributed to the effects of clinical and subclinical hypothyroidism in pregnancy [15]. The following values were assigned to each of the variables: *p*1 = 0.3 (ratio of adverse effects in the intervention group), *p*2 = 0.6 (ratio of adverse effects in the control group), *β* = 0.2, and power = 80%. Based on initial data, 42 subjects were calculated to participate in each group. Electrochemiluminescence (ECL) method and COBASS-E411 device were used to perform these tests. The defined CVs in the measurement kits were 3.2, 0.97, 2.48, and 5.9 for TSH, total T4, free T4, and anti-TPO Ab, respectively. Anti-TPO Ab levels of more than 34 IU were defined as a positive test. In addition to the predefined TSH level, other inclusion criteria included normal thyroid size without any nodules on examination, no history of thyroid surgery or previous administration of radioactive iodine or concomitant nonthyroid disease, and the presence of singleton pregnancy. The levothyroxine made by Iran Hormone factory, at least 50 micrograms daily, was used to treat patients. The TSH level was reevaluated 4 weeks after enrollment, then every 4 weeks until the 24th week of pregnancy, and finally at 32 to 36 weeks of gestation. Finally, 80 pregnant women met the inclusion criteria and were randomly assigned into two groups including the levothyroxine treatment group (*n* = 41) and nontreatment group (*n* = 39). Pregnant women who could not complete the follow-up period or did not use levothyroxine properly, those who developed another disease during pregnancy or had to take thyroid-metabolizing drugs such as corticosteroids and beta-blockers, were excluded from the study. The data obtained from this study were described using central and dispersion indices. We used chi-square and Fisher tests for qualitative variables, *T*-test for quantitative variables with normal distribution, and Mann–Whitney tests for quantitative variables without normal distribution. Data analysis was performed using SPSS software version 23.

## 3. Results

A total of 80 women with singleton pregnancies participated in the present study, of which 41 were placed in the intervention group and 39 in the control group. The youngest and the oldest participants were 16 and 43 years old, respectively, with a mean age of 28.79 years. There was no previous history of abortion in 75% and stillbirths in 97.5% of participants. Forty-three percent of women were considered nulliparous. The only qualitative variable that showed a significant difference between the two groups was the type of pregnancy (*p* = 0.023). The control group had a higher percentage of normal pregnancies compared to the intervention group. Regarding variables such as place of residence, mean maternal age, mean gestational age, history of fetal death, history of previous abortion, type of previous pregnancies, number of previous abortions, number of live births, number of deliveries, and history of hypothyroidism or gestational diabetes in previous pregnancies, there was no significant difference between the intervention and control groups (*p* > 0.05) (Table 1).

Among the quantitative variables, there was no significant difference in total T4, free T4, TSH, and anti-TPO Ab levels between the two groups. Although the amount of baseline TSH in the intervention group (3.4 mIu/L) was only slightly higher than that in the control group (3.2 mIu/L), this difference was considered statistically significant (*p* < 0.0001) (Table 2).

In the present study, the total frequency of positive anti-TPO Ab test (≥34 IU) was 20% (*n* = 15), of which 16.22% (*n* = 6) were considered in the intervention group and 23.68% (*n* = 9) in the control group. The total frequency of negative anti-TPO Ab test (<34 IU) was 80% (*n* = 60), of which 83.78% (*n* = 31) were considered in the intervention group and 76.31% (*n* = 29) in the control group (*p* value = 0.553).

The level of follow-up TSH (*p* = 0.018) and final TSH (*p* = 0.0001) of patients showed a significant difference between the two groups; however, changes in total and free T4 levels of patients during the follow-up period did not show a significant difference between the two groups (*p* = 0.444 and *p* = 0.375, respectively) (Table 3).

Among the adverse outcomes of pregnancy, placental abruption, placenta previa, eclampsia, preeclampsia, and neonatal death were not seen in our study. There was no significant difference between the intervention and control groups in terms of adverse pregnancy outcomes (*p* < 0.05). In total, 6.25% (5 women) lost their pregnancies. The rate of pregnancy loss in the intervention and control groups was 7.32% and 5.13%, respectively (*p* = 0.686). In this study, 10% of participants (8 women) had preterm delivery and its rate was the same in the intervention group as in the control group (*p* = 0.941). The rate of premature rupture of membranes did not show a significant difference between the intervention and control groups (*p* = 0.330). The most common complication observed in the control and intervention groups was preterm delivery with a percentage of 10.25 and 9.97, respectively (Table 4).

Fifteen women from all of the participants had a positive test for TPO Ab (≥34 IU). The rate of anti-TPO positivity was 3 in those who lost their pregnancies, while 12 in those who did not (*p* = 0.021). No association was found between anti-TPO Ab test positivity and premature rupture of membrane or preterm delivery (*p* > 0.05). Of the 39 participants in the control group, 9 participants, according to the level of follow-up TSH, reached a level beyond the Iranian recommendations for initiation of treatment with levothyroxine. Four out of these participants (44.44%), who needed treatment with levothyroxine, had a positive anti-TPO test (*p* = 0.008). Most of these women who needed treatment were in the second trimester of pregnancy (55.56%); however, this difference was not statistically significant in terms of time required for treatment (*p* = 0.059) (Table 5).

A subgroup analysis of women with positive anti-TPO Ab showed a significant more pregnancy loss in this group (*p* = 0.011). We did not find any meaningful statistical difference between the two groups about the first-minute low Apgar score of neonates. The frequency of first-minute low Apgar score in the intervention and control groups was 7.32 and 2.57%, respectively.

## 4. Discussion

This study showed that levothyroxine therapy in pregnant women with TSH levels between the numerical numbers of American and Iranian guidelines had no significant effect on adverse pregnancy outcomes including pregnancy loss, premature rupture of membranes, preterm delivery, eclampsia, and preeclampsia. However, in mothers who were positive for anti-TPO Ab, the need to start levothyroxine treatment during the follow-up period was higher than anti-TPO Ab-negative women. A cohort study from the United States showed an increased risk of miscarriage (OR = 3.66) when the TSH level in early pregnancy was above the 95th percentile. However, in this study, both subclinical and clinical cases of hypothyroidism were evaluated [16]. Our study did not show a significant difference in the incidence of abortion between the two groups. A retrospective study found that those who experience abortion during pregnancy had higher mean TSH levels and lower mean free T4 levels in early pregnancy [17]. Liu et al. reported an increased risk of miscarriage due to increased maternal TSH concentration, which was enhanced by the presence of a positive TPO Ab [6]. Negro et al. reported a higher rate of pregnancy loss in TPO Ab-negative women with TSH concentrations between 2.5 and 5 compared to those whose TSH concentrations were below 2.5 mIu/L (6.1% vs. 3.6%) [4]. Männistö et al. in another study showed that subclinical hypothyroidism increases the risk of pregnancy complications in TPO Ab-positive women [18]. Similarly, Benhadi et al. in a prospective cohort study of 2,497 Dutch women reported a higher risk of miscarriage, fetal death, or infant death in mothers with subclinical hypothyroidism [5]. In our study, a significant number of control members who had adequate TSH and T4 at the time of enrollment reached a range of TSH levels that required levothyroxine treatment in the second and third trimesters of pregnancy. A significant percentage of these subjects had positive anti-TPO antibody. This suggests that pregnant women who are at higher risk for thyroid diseases should have shorter intervals of hormonal follow-up, especially TSH level measurement, than others. Our study did not show a significant difference in the first-minute Apgar score of infants between the intervention and control groups. Feldthusen et al. showed a higher percentage of low Apgar scores at birth (less than 7 in 1 minute after birth) in pregnant women with subclinical hypothyroidism (TSH>4.4) compared to pregnant women with normal thyroid function (*p* = 0.02) [19].

Our study failed to establish a correlation between anti-TPO Ab positivity and preterm delivery or premature rupture of membranes. Korevaar et al. reported that higher levels of TSH and subclinical hypothyroidism increase the risk of preterm delivery. They also showed that anti-TPO Ab positivity was associated with a 1.7-fold increase in the risk of preterm labor and a 2.5-fold increase in the risk of very preterm labor [20]. In the present study, the most frequent complication of pregnancy was preterm delivery followed by pregnancy loss and the least complication was premature rupture of membranes. Although these findings were not significantly correlated to the TSH level of pregnant women, positive anti-TPO Ab mothers had a significantly higher rate of pregnancy loss and progress to the subclinical hypothyroid state. In the study of Liu et al. the complications of pregnancy, such as abortion at a much lower gestational age, were more than 2 times in anti-TPO Ab-positive women compared to TPO-Ab-negative ones [4–6]. Männistö et al. surveyed the association between pregnancy outcomes and thyroid function at 12-week gestation in 5805 pregnant women and found no significant association between thyroid function and prenatal morbidity [18]. The result of our study is consistent with the study of Männistö et al., but it does not agree with the results of the study by Liu et al. The reasons for the insignificance of the findings of our study were likely the small sample size and narrow range of TSH defined for the adverse consequences of pregnancy. Another reason for the difference in the results of this study with other studies could be the genetic difference between the different races in terms of susceptibility to the complications of any disease.

## 5. Conclusion

In pregnant women whose TSH range is between the two limits defined in the Iranian and American guidelines, lack of treatment with levothyroxine does not increase the adverse short-term consequences of pregnancy. Positive anti-TPO Ab may play a role in the development of short-term complications in mothers with subclinical hypothyroidism or it may increase the likelihood of an increase in TSH level during pregnancy.

## Figures and Tables

**Table 1:** Comparison of the frequency distribution of baseline qualitative characteristics between the intervention and control groups.

Variable		Intervention group (number (%))	Control group (number (%))	*p* value
Location	City	37 (52.1)	34 (47.9)	0.665
	Village	4 (44.4)	5(55.6)	

History of a dead fetus	Yes	0 (0.0)	2 (100)	0.23
	No	41 (52.6)	37 (47.4)	

Type of pregnancy	Normal	28 (44.4)	35 (55.6)	0.023
	IVF	6 (100)	0 (0.0)	
	Other cases (with medication)	7 (63.6)	4 (36.4)	

History of previous miscarriages	Yes	13 (65.0)	7 (35.0)	0.155
	No	28 (46.7)	32 (53.3)	

Pregnancy	First	17 (56.7)	13 (43.3)	0.738
	Second	15 (46.9)	17 (53.1)	
	Third and more	9 (50.0)	9 (50.0)	

Number of deliveries	None	25 (58.1)	18 (41.9)	0.146
	One	15 (53.6)	13 (46.4)	
	Two and more	1 (11.1)	8 (88.9)	

Number of previous abortions	None	30 (50.0)	30 (50.0)	0.597
	One	6 (45.2)	7 (53.8)	
	Two and more	5 (71.4)	2 (28.6)	

Number of live births	None	25 (58.1)	18 (41.9)	0.146
	One	15 (53.6)	13 (46.4)	
	Two and more	1 (11.1)	8 (88.9)	

History of gestational diabetes	Yes	2 (28.6)	5 (71.4)	0.209
	No	39 (53.4)	34 (46.6)	

Hypothyroidism in previous pregnancies	Yes	8 (80.0)	2 (20.0)	0.052
	No	33 (47.1)	37 (52.9)	

**Table 2:** Comparison of the baseline quantitative and clinical variables between the intervention and control groups (mean ± SD).

Variable	Intervention (*M* ± SD)	Control (*M* ± SD)	*p* value
Maternal age	28.1 ± 7.2	30.2 ± 5.3	0.163
Gestational age	15.27 ± 7.5	18.2 ± 8.4	0.104
TSH level	3.4 ± 0.30	2.3 ± 0.36	<0.0001
T4 level	10 ± 2.5	10 ± 2.4	0.90
Free T4 level	1.7 ± 3.1	1.5 ± 2.01	0.753

**Table 3:** Comparison of the follow-up hormones related to thyroid function between the intervention and control groups (mean ± SD).

Variable	Intervention (*M* ± SD)	Control (*M* ± SD)	*p* value
Follow-up total T4	10 ± 1.04	11.1 ± 3.09	0.444
Follow-up free T4	0.98 ± 0.35	0.86 ± 0.2	0.375
Follow-up TSH	2.06 ± 1.16	2.7 ± 1.2	0.018
Final TSH	1.64 ± 0.8	2.37 ± 0.86	0.0001

**Table 4:** Comparison of short-term pregnancy complications between the two groups.

Variable		Study group		Total	*p* value
		Intervention group (*n* (%))	Control group (*n* (%))		
Loss of pregnancy	Yes	3 (7.32)	2 (5.13)	5 (3.6)	0.686
	No	38 (92.68)	37 (94.87)	75 (8.93)	

Preterm	Yes	4 (9.75)	4 (10.25)	8 (0.10)	0.941
	No	37 (90.25)	35 (89.75)	72 (90.0)	

Premature rupture of the membranes	Yes	3 (7.32)	1 (2.56)	4 (0.5)	0.330
	No	38 (92.68)	38 (97.44)	76 (0.95)	
Total		41 (51.25)	39 (48.75)	80 (100)	

**Table 5:** Frequency of positive anti-TPO Ab in control cases with increasing TSH required to treatment with levothyroxine during the follow-up period according to Iranian guidelines.

Variable		Convert control group to treatment		Total	*p* value
		Yes (number (%))	No (number (%))		
Anti-TPO Ab positivity	Yes	4 (44.44)	26 (67.86)	30 (100.00)	0.008
	No	5 (56.55)	4 (33.13)	9 (100.00)	

Trimester of pregnancy	First	1 (11.11)	10 (33.33)	11 (20.28)	0.059
	Second	5 (55.56)	13 (33.43)	18 (15.46)	
	Third	3 (33.33)	7 (34.23)	10 (64.25)	

Total		9 (100.00)	30 (100.00)	39 (100.00)	

## Data Availability

The data used to support the findings of this study are included within the article.
